# Reassessing pre-existing comorbidity patterns in testicular cancer: controversies and future directions

**DOI:** 10.1093/hropen/hoaf079

**Published:** 2025-12-13

**Authors:** Yinwei Chen, Yi Liu, Chang Liu

**Affiliations:** Reproductive Medicine Center, Tongji Hospital, Tongji Medical College, Huazhong University of Science and Technology, Wuhan, China; Reproductive Medicine Center, Tongji Hospital, Tongji Medical College, Huazhong University of Science and Technology, Wuhan, China; Reproductive Medicine Center, Department of Obstetrics and Gynecology, West China Second Hospital, Sichuan University, Chengdu, China; Key Laboratory of Birth Defects and Related Diseases of Women and Children, Sichuan University, Ministry of Education, Chengdu, China

Dear Editor,

We read with great interest the recent article published in *Human Reproduction Open* by Ornstrup *et al.* (2025), which investigated pre-existing morbidity patterns in testicular cancer (TC) patients using the nationwide prospective clinical Danish Testicular Cancer (DATECA) database. They found that the increased hospital contacts and medicinal use in TC patients prior to diagnosis provided valuable insights into the potential etiological links. However, three critical concerns should be further clarified to strengthen the validity of the conclusions.

First, the control group lacks objective verification of testicular health, raising the risk of undiagnosed TC contamination. The authors enrolled controls solely based on the absence of TC registration in the DATECA database, which enabled the above-stated hypothesis to be possible. The fact that 49 participants were excluded from the control group for the registration of TC in other databases (National Patient Registry and National Center Registry) also supports our theory. The reliance on administrative data overlooked the inherent limitations of registry-based studies: early-stage TC or asymptomatic metastases might remain undiagnosed due to low medical-seeking behaviors, socioeconomic barriers, or lack of routine testicular examination. Supplementing clinical data, such as scrotal ultrasound findings and serum tumor markers (β-hCG, lactate dehydrogenase (LDH), alpha-fetoprotein (AFP)), which exclude TC diagnosis in the control group, would enhance the internal validity of the cohort comparison.

Second, unadjusted confounding factors may participate in the association between TC and multi-system morbidity. Ornstrup *et al.* (2025) reported that patients presented with significantly increased morbidity across multiple health domains before the diagnosis of TC, whereas this phenomenon might be derived from shared risk factors rather than direct biological links between TC and other disorders. Well-established TC risk factors, including long-term smoking (Dusek *et al.*, 2008), perfluoroalkyl and polyfluoroalkyl substances (PFAS) exposure (Calvert *et al.*, 2021), and occupational contact with organic solvents (Guth *et al.*, 2025), are also critical contributors of the comorbidities (e.g. respiratory (GBD 2019 Tobacco Collaborators, 2021), neurological (Gerhardsson *et al.*, 2021), and urogenital systems (O’Sullivan Bakshi *et al.*, 2025)) that they observed in the TC group. This study failed to collect relevant data to adjust for confounding exposures, which limited its conclusion attributing the links between TC and multi-system morbidity to potential pathophysiological mechanisms. Variables like lifestyle factors, environmental exposures, and occupational history should be included in future analyses.

Third, the exception for hospital contacts and medicine use, which is limited to 90 days prior to TC diagnosis, is inadequate. To minimize capturing healthcare use directly related to the imminent TC, Ornstrup *et al.* (2025) excluded hospital contacts and drug prescriptions within 90 days before the diagnosis. According to Chalya *et al.* (2014), the median time from symptom onset to TC diagnosis is 8 months (range: 2–16 months) During this pre-diagnostic period, patients may seek medical assistance for TC-related symptoms (e.g. scrotal discomfort, breast development, fatigue, etc.), which would be incorrectly classified as pre-existing comorbidity. This issue is further emphasized by the relatively high metastasis rate (20.7%, 404/1952) at diagnosis in the TC cohort. TC commonly metastasizes to the lungs, distant lymph nodes, bones, liver, and brain (Shah *et al.*, 2023). Symptoms from these metastatic lesions (such as a cough from lung metastases) can manifest for months before the primary cancer is diagnosed. Thus, extending the exception period, which aligns with TC’s diagnostic timeline and reduces the risk of conflating metastatic disease with pre-existing comorbidity, might be more reasonable.

We appreciate Ornstrup *et al.*’s (2025) valuable contribution to understanding pre-existing morbidity patterns in TC patients. Addressing these concerns would not only strengthen the study’s conclusions but also help clinicians to distinguish TC-related manifestations and other comorbidities, guiding future research into shared etiological factors.
